# From Single Target to Multitarget/Network Therapeutics in Alzheimer’s Therapy

**DOI:** 10.3390/ph7020113

**Published:** 2014-01-23

**Authors:** Hailin Zheng, Mati Fridkin, Moussa Youdim

**Affiliations:** 1 Department of Medicinal Chemistry, Intra-cellular Therapies Inc. 3960 Broadway, New York, NY 10032, USA; 2 Department of Organic Chemistry, Weizmann Institute of Science, Rehovot 76100, Israel; E-Mail: mati.fridkin@weizmann.ac.il; 3 Abital Pharma Pipeline Ltd., Tel Aviv 6789141, Israel; E-Mail: youdim@tx.technion.ac.il

**Keywords:** memantine, nitromemantine, NMDA antagonist, M30, M30D, AChE-MAO-A/B inhibitor, chelator, *Ginkgo biloba*

## Abstract

Brain network dysfunction in Alzheimer’s disease (AD) involves many proteins (enzymes), processes and pathways, which overlap and influence one another in AD pathogenesis. This complexity challenges the dominant paradigm in drug discovery or a single-target drug for a single mechanism. Although this paradigm has achieved considerable success in some particular diseases, it has failed to provide effective approaches to AD therapy. Network medicines may offer alternative hope for effective treatment of AD and other complex diseases. In contrast to the single-target drug approach, network medicines employ a holistic approach to restore network dysfunction by simultaneously targeting key components in disease networks. In this paper, we explore several drugs either in the clinic or under development for AD therapy in term of their design strategies, diverse mechanisms of action and disease-modifying potential. These drugs act as multi-target ligands and may serve as leads for further development as network medicines.

## 1. Introduction

During the past two decades, drug discovery has mainly focused on the single-target paradigm (single keys for specific locks), pursuing exquisitely selective ligands to drug targets with the hope of avoiding unwanted side effects. Thanks to the advances in molecular biology that enable researchers to define a key target for a particular disease, this paradigm has achieved considerable success and will continue being pursued in the future. However, the single-target drugs are limited in the treatment of complex diseases such as cancer, depression, and Alzheimer’s disease (AD), because complex diseases have multiple pathogenic mechanisms and are not likely to result from a single defect. Attempts to develop more effective treatments for complex diseases by discovering highly selective drugs have been largely unsuccessful [1]. Taking anti-AD drugs as an example, in the past 10 years, none of the developing anti-AD drugs survived Phase 3 clinical trials due to the lack of efficacy in improving the cognitive deficits in AD patients [2].

Network medicines may offer alternative hope for effectively fighting against AD and other complex neurodegenerative disorders. Advances in medical biology suggest that diseases, especially complex diseases, are caused rarely by a single gene abnormality, but most likely by the perturbations of the complex intracellular and intercellular network that links tissue and organ systems. Network medicine contrasts the prevailing scientific reductionism that relies on targeting single proteins (enzymes)/mechanisms to provide therapeutic effects to complex diseases. Instead, network medicine approach views diseases as specific types of network perturbation due to activation or suppression of certain stages. It employs more holistic approaches to restore network perturbation by simultaneously targeting key components in disease networks [3]. From the network-based view, most disease phenotypes are difficult to reverse through an intervention affecting a single node in the network, that is, an action of a single-target drug. This could be explained in term of the feedback mechanisms and the robust phenotypes of biological systems [4]. Because there exist the feedback mechanisms in biological systems, a long-term enzymatic inhibition may not be able to decrease, but instead may increase the enzyme activity. For example, it has been reported that the rapidly-reversible acetylcholinesterase (AChE) inhibitors (donepezil, galantamine and tacrine), which were intended for decreasing AChE activity, were found to significantly increase AChE activity and protein levels in the cerebrospinal fluid (CSF) of AD patients after long-term (1 year) treatment [5]. This may partly contribute to the limited efficacy of these AChE inhibitors in AD treatment. Robustness is another key factor that prevents single-target drugs from exerting their therapeutic effects. As an intrinsic property of biological systems, robustness can derive from the structure of biological network and results from compensatory routes that bypass the inhibition of individual proteins. Thanks to this intrinsic property, our bodies can maintain their functions in the face of various attacks and perturbations. But, it is also because of the robustness in our bodies that make drugs, in particular, single-target drugs, less effective or even ineffective [4]. However, network biology also suggests that although, in most cases, deletion of individual nodes (genes or proteins) may have little effect on disease networks, simultaneous modulation of genes/proteins can perturb or even destroy the robust phenotype in living systems. For example, experiments have demonstrated that simultaneous deletion of two genes can lead to synthetic sickness or synthetic lethality although cells with either of the single gene deleted are viable with no sick feature [6,7,8]. In fact, these principles have been successfully applied to drug development in cancer, HIV, and antibacterial therapy, in which simultaneous modulation of two or more targets, either using multi-target drugs or combination therapy, to achieve better therapeutic efficacy has became daily practice [9].

In addition, network models suggest that partial inhibition of a carefully selected small number of targets can be more efficient than complete inhibition of a single well selected target, with potential less side effects. Indeed, the development of multi-target antidepressants has provided a set of proof-of-principle experiments to support this concept. In Alzheimer’s field, recent studies have demonstrated that simultaneous moderate inhibition of both BACE1 and γ-secretase was found effective and safe in AD mice, with no evidence of toxicity, while completely knocking out BACE1 or γ-secretase led to serious side effects [10]. Studies have also shown that synergistic combination therapy tends to improve therapeutically relevant selectivity, and can achieve desired therapeutic efficacy with decreased doses of each drug by overcoming compensatory mechanisms, thereby minimizing toxicity and other side effects related to high doses of single drugs [11].

Although multi-target drugs are not true network medicines yet, they may be considered as simplified versions or lead drugs that could be further developed into network medicines. We believe that network medicines have a great promise for offering alternative hope for effectively fighting against AD and other complex diseases. In this paper, we explore some selected multi-target drugs, either in clinic or in development for AD therapy, which have potential for developing as network medicines.

## 2. Memantine, An Anti-AD Drug that Breaks Conventional Rules

Excessive activation of glutamate receptors, specifically *N*-methyl-d-aspartate receptors (NMDARs) has been implicated in the pathophysiology of several neurodegenerative diseases including AD and Parkinson’s disease (PD). In comparison with non-AD state, glutaminergic neurons in AD show overactivation, releasing more and continuously glutamate. The excessively released glutamate stimulates glutamate receptors, specifically NMDARs, causing high levels of calcium ions (Ca^2+^) to influx into the postsynaptic cell. This excess Ca^2+^ influx into cells activates a number of enzymes that damage cell structures (cytoskeleton, membrane and DNA), leading to neuronal death. Besides having a role in direct neuronal death, hyperactive NMDARs have also been reported to increase tau hyperphosphorylation contributing to neurofibrillary tangles, which is related to neurofibrillary degeneration and tau toxicity in AD [12,13]. In addition, there are several potential links between excitotoxic damage and Aβ toxicity. For example, soluble Aβ oligomers can significantly decrease synaptic glutamate uptake to perturb synaptic plasticity and promote synapse depression [14]; Aβ peptide can bind directly to glutamate receptors to increase the receptor activity, leading to elevated intracellular Ca^2+^ and consequent excitotoxicity [15,16]; and also Aβ-related peptides can potentiate potassium-evoked glutamate release from adult rat hippocampal slices and cortex [17].

Due to the critical role of glutamate in the pathophysiology of AD and other neurodegenerative diseases, targeting glutamate receptors have been considered as a good therapeutic intervention for many years. Indeed, a large number of such antagonists have been developed based on the conventional paradigm “high affinity/high specificity”, which dominates the drug discovery in the pharmaceutical industry during the past decades. However, all such antagonists except memantine have disappointingly failed in advanced clinical trials, in large part because of unacceptable side effects [18]. Memantine is the first and only NMDARs antagonist up-to-date approved by FDA for the treatment of moderate-to-severe AD and in dementia with Lewy bodies. In three key double-blind, placebo-controlled trials, mementine showed statistically significant, clinical benefits on cognition, function, and global status for patients with moderate to severe AD. Data from 27 clinical trials and over 600,000 patients demonstrated that mementine was safe and well-tolerated with a safety profile similar to that of placebo treatment [19].

The successful introduction of memantine into markets for AD treatment has profound implications for drug discovery. The well-tolerated safety profile of memantine in contrast to the severe side effects of other similar NMDARs antagonists has aroused extended research on its mechanisms of action. It is now known that glutamate is a key transmitter that mediates the normal physiological processes in excitatory synaptic transmission, which is critical for plastic synaptic changes including long-term potentiation, memory, and learning. Drugs with high affinity binding to NMDARs are expected to bind too tightly and end up blocking virtually all receptor activation including normal receptor activation, consequently leading to clinically unacceptable side effects [18]. One such example is dizocilpine (MK-801), a potent NMDAR antagonist with K_i_ of 30.5 nM [20]. Studies have demonstrated that dizocilpine, due to its high affinity binding to NMDARs, blocked not only excessive NMDAR activity, but also crucial normal physiological activity in all parts of the brain. This has been considered as the underlying mechanism of the drug’s side effect. By contrast, memantine possesses surprising low-affinity binding to NMDARs with an IC_50_ of about 1 μM. It has almost no selectivity among subtype NMDARs with IC_50_ values of 0.89, 0.40, 0.32 and 0.28 μM for NR2A, NR2B, NR2C and NR2D receptors expressed in X. oocytes, respectively [21].

Memantine has a weak potency at the NMDARs, with poor selectivity between subtype receptors. Thus, it was initially regarded as a poor drug candidate for AD therapy. Indeed, the successful story of memantine for AD therapy breaks all the conventional rules of screening high-affinity competitive antagonists for their targets, and has greatly influenced the neuroprotective drug development in the future. The mechanisms by which memantine exerts its clinical benefits with safe profile have attracted a great interest in the field of medicinal chemistry [18].

### 2.1. Mechanisms of Action of Memantine

Accumulating evidence has indicated that memantine can bind to several targets with relatively low-affinity and possesses multiple neuroprotective activities in various cell and animal models.

#### 2.1.1. Low-affinity, Dirty and Uncompetitive NMDA Antagonist

Multiple mechanisms of action may contribute to the therapeutic efficacy of memantine in the clinic, however, its principal mechanism of action is believed to be the blockade of excessive NMDAR activity. It has been well documented that memantine is a voltage-dependent, low affinity, uncompetitive NMDAR antagonist with fast on/off kinetics. It inhibited NMDARs with an IC_50_ of about 1 μM, and with almost no selectivity among subtype NMDARs. The well-tolerated and safe profile of memantine in the clinic in contrast to other NMDAR antagonists can be attributed to the drug unique characteristics: low-affinity, fast on/off kinetics and uncompetitive antagonism. It is now understood that it is because of the low-affinity along with its unique fast on/off kinetics that make memantine distinguish from other high-affinity NMDAR antagonists that failed in clinical trials. NMDAR antagonists with high-affinity are expected to bind tightly to their receptors and block virtually all receptor activity, leading to severe side effects. By contrast, drugs with low-affinity will not bind very well under physiological conditions so as to spare normal physiological activity, but can selectively block excessive activity of NMDARs under pathological conditions. In addition, the fast on/off kinetics of memantine allows it to sit on the receptor just long enough to suppress the pathologic activation, while off the receptor fast enough to prevent the drug accumulating in the ion channels. This unique property helps prevent interference with normal synaptic transmission. The uncompetitive antagonistic effect of memantine also plays an important role in the drug’s safe profile. Competitive antagonists (especially high-affinity antagonists), which compete with their agonists’ binding sites, likely block all the NMDARs’ activities, including both physiological and pathological activities. Moreover, these types of antagonists work better to block normal function than pathological activity, and thus lead to clinically unaccepted side effects [18,22].

#### 2.1.2. Effects on Other CNS Targets

Besides acting as a NMDAR antagonist, memantine can also bind to many other CNS targets and modulate their activities. For example, it can bind to different neuronal nicotinic acetylcholine receptors (nAChRs) at potencies possibly similar to the NMDA receptors. But the reported potency of memantine at α7 nicotinic acetylcholine (ACh) receptors varies considerably, with the IC_50_ value of 5 μM at human receptors in *Xenopus* oocytes and 0.33–1.68 μM at rat receptors [23,24]. Memantine also blocked human 5-HT_3_ receptors stably expressed in HEK-293 cells and native murine 5-HT_3_ receptors in the N1E-115 cell line. It acted as a non-competitive antagonist at the 5-HT_3_ receptor, with potency similar to that for the NMDA receptor *in vitro* in a non-use, non-voltage-dependent manner [25]. Furthermore, studies also showed that memantine possessed agonist action at dopamine D2^High^ receptors with similar or greater potency than that at the NMDA receptor [26]. This may have considerable clinical relevance, as D2^High^, the high affinity state of the D2 dopamine receptor, is the functional state of dopamine D2 receptor [27,28].

#### 2.1.3. Neuroprotective Activity in Various Culture and Animal Models

In addition to modulating different CNS targets, memantine also exhibits a large variety of neuroprotective activities in numerous culture and animal models, including protection against Aβ toxicity, tau phosphorylation, neuroinflammation, and oxidative stress.

Early studies demonstrated that memantine blocked the toxic effects of Aβ_1−40_ in cultured cortical neurons and attenuated Aβ_1−42_-induced reduction of neurite outgrowth in neuronal cultures [29,30]. In addition, memantine, at therapeutically relevant concentrations, also protected against neuronal loss and apoptosis induced by the direct injection of Aβ_1−40_ into the hippocampus [31]. More recently, memantine was shown to lower levels of secreted APP and Αβ_1–40_ in human neuroblastoma cells at therapeutically relevant concentrations (1–4 μM) and reduce Aβ_1–42_ production at subtoxic concentrations (4 and 18 μM) in rat primary cortical cultures. In APP/presenilin-1 (PS1) transgenic mice with high brain levels of Aβ_1–42_, memantine treatment (20 mg/kg/day p.o. for 8-day) significantly reduced the brain levels of soluble Aβ_1–42_ [32]. In a Tg2576 mouse AD model, long-term (6 months) administration of memantine (5, 10 and 20 mg/kg/day) was found to significantly decrease Aβ plaque deposition, increase synaptic density, with two higher doses of memantine (10 and 20 mg/kg/day) associated with a significant increase in degenerating axons [33]. Other studies reported that memantine treatment reduced the total cortical levels of membrane-bound amyloid precursor protein (45%–55%) in both transgenic and nontransgenic mice, which eventually may decrease the Aβ level [34].

The effects of memantine on tau phosphorylation have also been reported, in which memantine protected rat cortical cultured neurons against Aβ-induced toxicity by attenuating tau phosphorylation [35]. Consistent with this, more recent studies showed that in primary mouse cortical neurons, Ca^2+^/calmodulin-dependent protein kinase β (CaMKKβ) activation of AMPK in response to Aβ_1−42_ led to increased phosphorylation of tau at Ser262/Ser356 and Ser396, which was blocked by memantine [36]. In early studies, memantine was found to inhibit and reverse tau accumulation in organotypic culture of rat hippocampal slices and abnormal hyperphosphorylation caused by protein phosphatase (PP)-2A inhibition *in vitro* [37]. *In vivo*, the CSF levels of phosphorylated tau in advanced AD patients treated with memantine for 1 year showed a statistically significant reduction, while non-phosphorylated tau and Aβ remained unchanged [38].

Inflammatory processes are also thought to contribute to the neurodegenerative changes during AD pathogenesis. In an animal model of neuroinflammation, memantine (at the therapeutically relevant dose in rats of 20 mg kg^−1^ day^−1^ s.c.) provided significant neuroprotection against the cytotoxic effects of proinflammagen lipopolysaccharide (LPS)-induced neuroinflammation on cholinergic neurons [39]. Consistent with this, other studies reported that low, therapeutically relevant doses of memantine (10 mg/kg/day, for 28 days) protected against LPS-induced neuroinflammation and restored behaviorally-induced gene expression and spatial learning in the rat [40]. Recent studies suggested that memantine exerted its anti-inflammatory effect by reducing microglia-associated inflammation and by stimulating neurotrophic factor release from astroglia, which is not related to and far from the NMDA receptors [41].

Oxidative stress is also a major aspect of AD pathology. Methylmercury (MeHg) has been shown to cause long-lasting neurological deficits in animals and humans. *In vivo* with a rat model, MeHg administration (12 μmol/kg MeHg for 4 weeks) was found to disrupt glutamate metabolism, induce oxidative damage, and increase neuron apoptosis in cerebral cortex. Pretreatment with memantine at a dose of 5 μmol/kg significantly prevented MeHg-induced alterations of Glu metabolism and oxidative stress, and alleviated neuron apoptosis [42]. Soluble oligomers, which include low molecular-weight (LMW) and high molecular-weight (HMW) species, are increasingly recognized as key mediators of synaptic and cognitive dysfunction in AD [43]. Recent studies reported that memantine can rescue both neuronal oxidative stress and transient memory impairment caused by HMW oligomers, but had no effect on the persistent memory deficit induced by LMW oligomers [44]. As both LMW and HMW oligomers can exert toxic effects on neurons, and their combined toxicity translates into the classical clinical symptoms of AD including persistent cognitive impairment, the reported results may provide a mechanistic explanation for the limited efficacy of memantine in preventing memory loss in AD [43].

**Figure 1:**
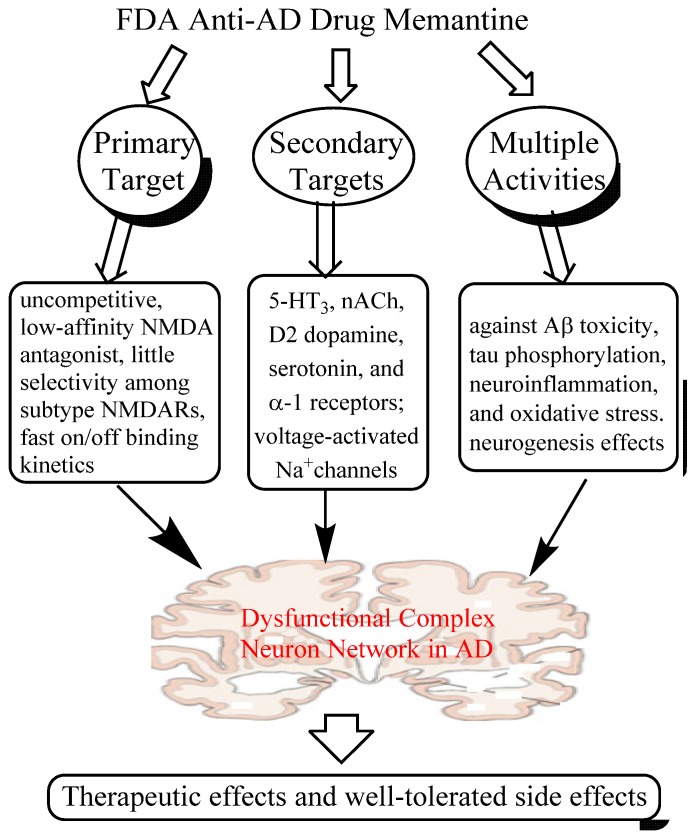
A system network view of memantine action: memantine interacts with multiple primary and secondary targets and exerts its manifold neuroprotective effects, which may synergistically contribute to its therapeutic effects and well-tolerated profile in the clinic.

Taken together, it is clear that memantine is a multi-target drug, acting primarily as an antagonist to block excessive activity of NMDARs, but also affecting several other CNS targets and exhibiting multiple neuroprotective properties in various models. All these activities may synergistically contribute to the therapeutic efficacy and safety profile of the drug in the clinic. In particular, the neuroprotective effects of memantine may play an important role in its disease-modifying potential in AD patients, in which meta-analysis of several trials suggests that memantine has potential to reduce clinical worsening and slow down the right hippocampal atrophy [45,46]. More importantly, the mechanisms of action of memantine can provide a rationale for scientists to design more effective multi-target ligands or network medicines for AD therapy. The multiple CNS targets and several key activities of memantine are summarized in Figure 1.

## 3. Nitromemantine: Second Generation Memantine Analog

**Figure 2:**
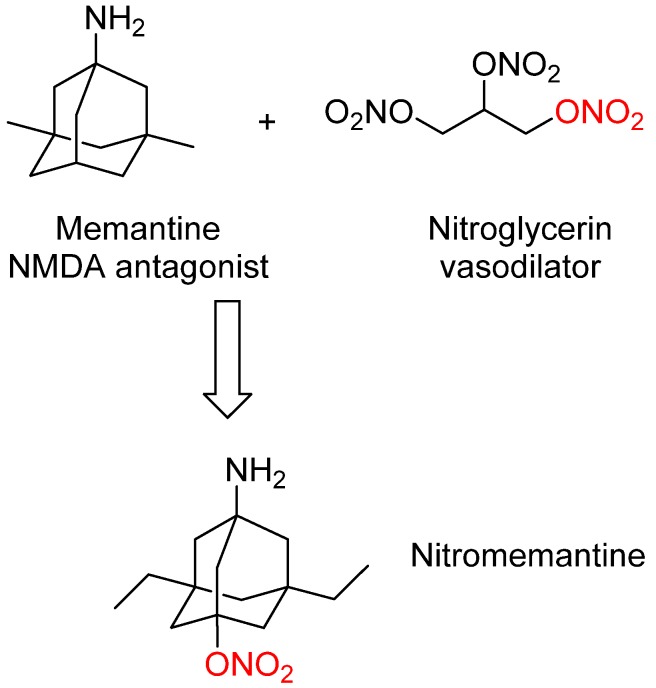
Design strategy leading to second generation memantine analog nitromemantine, in which the important pharmacophore (nitrooxy moiety red–ONO_2_) from the FDA-approved drug nitroglycerin was introduced into the structure of memantine.

Memantine has shown efficacy in the clinic for treating moderate to severe AD with some potential disease-modifying effects. In order to improve memantine efficacy and endow it with significant disease-modifying activity, second-generation memantine analogs were developed by Lipton’s group [18]. These novel analogs were designed by connecting memantine with another important pharmacophore (nitrooxy moiety –ONO_2_) from another FDA-approved drug nitroglycerin (Figure 2). Nitroglycerin is one of the oldest and most useful drug for treating and preventing attacks of angina pectoris. *In vivo* nitroglycerin can be converted to nitric oxide (NO) that can react with cysteine thiol groups to form S-nitrosothiols, which is termed as S-nitrosylation. S-nitrosylation can change protein function, producing either neuroprotective or neurodestructive effects depending on the proteins involved [47,48].

Previous studies have shown that there exists a nitrosylation site on the extracellular domain of NMDA receptor, and S-nitrosylation of this site can decrease excessive receptor activity, positively modify neuronal survival, and block apoptotic cell death, and therefore offering neuroprotection to sick neurons [48]. However, neither nitroglycerin nor nitric oxide is a good neuroprotectant for AD therapy because, if administered systemically, they can cause serious side effects (such as severe hypotension) and may even be toxic due to the reaction of nitric oxide with superoxide anion (O^2−^) to form peroxynitrite (ONOO^−^). To avoid these systemic side effects, nitric oxide-releasing moiety (nitrooxy moiety –ONO_2_) from nitroglycerin was attached to memantine to create new bifunctional drugs. The rationale of this design was based on the facts: (1). The NMDAR has other modulatory sites and S-nitrosylation of these sites is neuroprotective, which could offer safe but effective clinical intervention; (2). As memantine rather selectively binds to the NMDAR, it could also function as a vehicle to target NO to the receptor; (3). The new memantine analogs may possess similar safety profile as memantine with improved efficacy and even disease-modifying effects.

Nitromemantine is the lead identified from these second-generation memantine analogs, which is expected to possess more neuroprotective efficacy than memantine without sacrificing clinical tolerability. Indeed, recent studies, both *in vitro* and *in vivo* in animal models, have demonstrated the feasibility of this novel drug strategy. *In vitro*, mixed neuronal/astrocytic cultures exposure to oligomerized Aβ_1−42_ or micromolar Aβ_25−35_ led to an increase in neuronal Ca^2+^ levels and NO formation. Both memantine and nitromemantine were found to largely abrogate the increase in Ca^2+^ levels and prevent the toxic NO production, but nitromemantine showed more effective in both cases. These results are consistent with previous reports that activation of extrasynaptic NMDARs is involved in Aβ-induced increase in neuronal Ca^2+^ and NO, and nitromemantine is more effective than memantine in blocking the activation of extrasynaptic NMDARs [49,50]. In hippocampal slices, oligomeric Aβ was found to induce extrasynaptic NMDAR mediated synaptic depression. This depression was inhibited either by 10 μM memantine or 5 μM nitromemantine at the same conditions, which suggests the greater potency of nitromemantine at extrasynaptic NMDARs over memantine [51].

NMDARs exhibit paradoxical effects on neurons, either promoting neuronal health or killing neurons, which depends on the receptor location. Synaptic NMDARs activity stimulates neuroprotective transcriptional and antioxidant pathways, whereas extrasynaptic NMDARs activity promotes cell death [52]. For example, synaptic NMDAR activity was found to reduce the levels of tau and phospho-tau (p-tau) in mixed neuronal/glial cultures, whereas extrasynaptic NMDAR activity exhibit opposing effects. Selective blockade of extrasynaptic NMDAR activity using either memantine or nitromemantine (5 μM each) decreased both p-tau and total tau levels induced by exposure to oligomerized Aβ_1−42_ *in vitro*. In addition, extrasynaptic NMDARs activity was found to mediate Aβ-induced molecular cascades leading to synaptic spine loss. Either memantine or nitromemantine inhibited eNMDARs preferentially over sNMDARs activity, significantly ameliorating the effects of Aβ on synaptic loss. However, in all these cases, nitromemantine displayed a larger and more significant protection than memantine. *In vivo*, the effects of memantine and nitromemantine on extrasynaptic NMDARs in a triple transgenic (3 × tg) AD mouse model were evaluated. The triple transgenic (3 × tg) AD mice exhibit deficits in synaptic function and cognition that occurs prior to extracellular Aβ deposition and tangles, but is associated with intracellular Aβ immunoreactivity. In 9-mo-old 3 × tg AD mice, nitromemantine demonstrated greater effects than that of memantine on increasing synaptic and dendritic density. In addition, only the nitromemantine-treated group showed significantly improved function on the location-novelty recognition test. Moreover, these experiments showed that nitromemantine was able to reverse the loss of brain connections and brings the number of synapses all the way back to normal within a few months of treatment in mouse AD models. This suggests that nitromemantine may have disease-modifying effect. And also, nitromemantine did not manifest any ill effect on blood pressure [18,51]. Based on these exciting results in animal models, nitromemantine is now advancing to human trials, bringing new hope to both early and later-stage Alzheimer’s patients.

## 4. Drugs Targeting Both AChE and NMDA Receptors

Acetylcholinesterase Inhibitors (AChEIs) are the first FDA-approved drugs for AD treatment, which are believed to exert their therapeutic benefits by increasing cholinergic levels. In the clinic, AChEIs such as donepezil and galantamine have been found to improve cognition and global behaviors in AD patients, although the effects are symptomatic with no significantly affecting the disease progression. These AChEIs were initially designed to act as single-target drugs to inhibit AChE, but accumulating evidence suggests that multiple effects may synergistically contribute to their therapeutic efficacy [53].

Since both cholinergic and glutamatergic dysfunction accounts for AD pathogenesis and the AChEIs do not display significant activity to antagonize over activation of NMDARs, network system biology suggests that combination therapy with memantine and AChEIs may have additive or even synergistic efficacy in AD treatment. They may simultaneously modulate both glutamatergic and cholinergic neurotransmitter systems. Indeed, patients treated with both memantine and AChEIs have shown greater efficacy over monotherapy and significantly slowed the rate of disease progression with good safety and tolerability [54]. And also, the addition of memantine to stable donepezil therapy in patients with moderate AD, and in those with moderate to severe AD, also significantly reduced 24-week decline in cognition, function and global status. The observed benefits were over and above those treated with donepezil alone [55].

**Figure 3:**
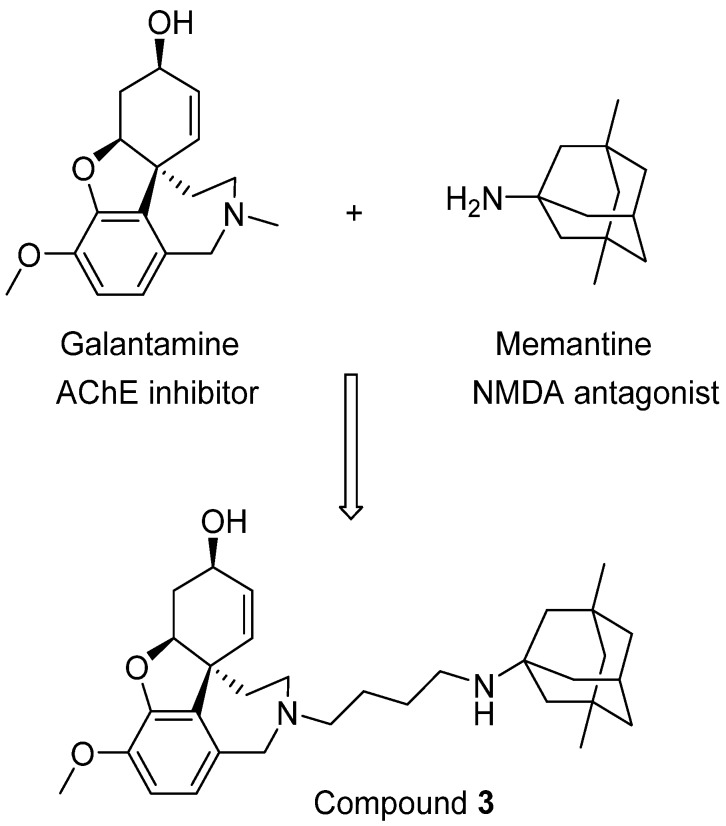
Linking two FDA-approved drugs leads to a new bifunctional drug candidate.

Moving forward this combination therapy is to create new neuroprotective molecules with activity against both AChE and NMDARs, which can be termed as bi/multi-target drugs. Several such attempts have been reported, and one such example was designed by the groups of Rosini and Cavalli [56]. In their research, a novel series of dual inhibitors of AChE and NMDARs were created by linking together two FAD-approved drugs galantamine and memantine (Figure 3). Among these new inhibitors, compound **3** was found to be the most potent NMDAR antagonist with a Ki of 2.32 μM while also possessing potent activity against AChE (IC_50_ = 0.696 μM). Although it is still difficult to balance the affinity profiles against both AChE and NMDARs, the interesting biological profile obtained from their research deserves further investigation [56].

## 5. M30: Metal/MAO Network Dysfunction Modulator

Brain functional network in AD patients is disrupted, which involves many proteins (targets) and pathways. Some of key disease-related targets/pathways include Aβ peptide, tau phosphorylation, metal dyshomeostasis, oxidative stress, and MAO dysfunctions in the brain. These dysfunctions are closely associated with the two characteristic hallmarks of AD: Aβ plaques and neurofibrillary tangles (NFTs) [53]. Substantial evidence has suggested that metal chelators or metal protein attenuating compounds (MPACs), antioxidants, and monoamine oxidase inhibitors (MAOIs) all have the potential as anti-AD drugs, and have also been investigated in the clinic [53]. For example, clioquinol (CQ), a MPAC, has been tested in phase II clinical studies for AD therapy. It significantly decreased the rate of cognitive decline in moderately severe AD patients when given orally (125–375 mg/twice daily over 36 weeks). However, the clinical trials were discontinued because CQ was suspected to be related to subacute myelooptico-neuropathy (SMON) [57].

The MAO-B inhibitor selegiline is a drug that is mainly used for the treatment of Parkinson’s disease. High dosages of selegiline, which inhibit both MAO A and B, are used in veterinary medicine to treat cognitive dysfunction (Canine Cognitive Dysfunction) in dogs [58]. In animal models selegiline was found to improve learning and memory deficits associated with AD, and slow the disease progression in AD patients with moderately severe impairment [59]. However, despite this initial promise, the efficacy of selegiline for AD therapy remains controversial because the effects were observed in some trials and the lack of benefit in several other trials [60].

Antioxidants such as vitamin E have been investigated both in animal models and in human as a potential drug for AD therapy. In mouse models, vitamin E supplementation in young mice was found to reduce lipid peroxidation, Aβ levels and amyloid deposition [61]. In human trials, vitamin E treatment (1350 mg/per day, 2 years) in 341 patients with moderately severe AD showed significant delays in the disease progression [59]. In a subsequent clinical trial, however, vitamin E treatment in patients with mild cognitive impairment (MCI) did not show significant efficacy in modifying the AD progression [62].

The controversial results from selegiline, vitamin E, and other similar studies suggest that modulation of a single target/pathway among the multiple disease-related targets/pathways in AD may not sufficient to restore the network dysfunction in AD brain and exhibit efficacy in the clinic. It is known that AD brain contains high levels of transition metals such as iron and copper, which could catalyze continual production of free radicals through the Fenton or Haber–Weiss reactions. This could undermine the efficacy of antioxidants to reduce oxidative damage and counteract the benefits of antioxidants in the clinic [63].

**Figure 4:**
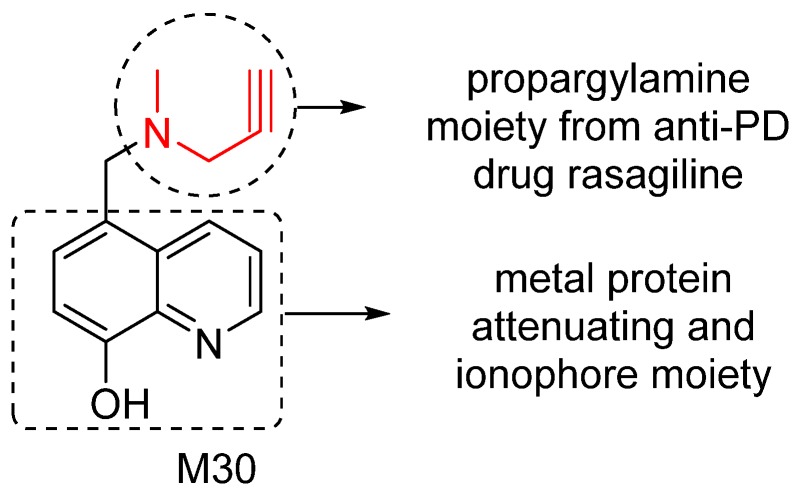
M30 structure and important pharmacophores.

To effectively restore the network dysfunction related to metal homeostasis, oxidative stress and MAO activity in AD brain, we have designed and investigated a series of new compounds as potential multitarget/network medicines. Among them, M30 was identified as a lead compound for further development. M30 contains a key pharmacophores (propargylamine moiety) from the FDA-approved anti-PD rasagiline (Figure 4). Rasagiline is a new generation of MAO-B inhibitor used as monotherapy or adjunct therapy to levodopa for early and late PD patients. In addition, rasagiline was recently reported to be the first drug with an apparent disease-modifying effect, at a dose of 1 mg per day [64]. The propargylamine moiety in the structure of rasagiline plays a pivotal role in the disease-modifying potential of rasagiline because it mediates the interaction of rasagiline with an array of neuroprotective/neurorescue pathways [65].

M30 also contains a metal protein attenuating/ionophore moiety (shown in Figure 4) similar to that of PBT2, a second generation MPAC that now is under Phase 2 clinical trials for AD treatment [66]. MPACs usually display intermediate reversible affinity towards metals and have potential to reach specific intracellular compartments to target the harmful “up-stream” metal-protein reactions. Rather than systemic binding and removal of metals from tissues (with potential systemic toxicity), MPACs restore metal dyshomeostasis, block free and reactive coordination positions of protein bound metals, and abolish specific and deleterious metal–protein interactions. Therefore, they hold promise as anti-AD drugs for slowing and/or preventing the disease progression [67].

Accumulating studies have shown that M30 is a multi-target ligand that may be further developed as a network medicine for AD treatment. *In vitro*, M30 irreversibly inhibited both MAO-A and B with IC_50_ values of 0.037 ± 0.02 μM and 0.057 ± 0.01 μM for MAO-A and -B, respectively [68]. *In vivo*, M30 showed brain selective inhibition for MAO-A and -B activity with relatively poor inhibition of these enzymes in the liver and small intestine [69]. This suggest that M30 may also have antidepressant activity by increasing brain levels of dopamine (DA), noradrenaline (NA), and serotonin (ST) with limited potentiation of tyramine pressor effect. Indeed, recent studies have shown that rats treated with M30 following oral administration of tyramine showed limited potentiation of blood pressure, similar to that of moclobemide, a selective reversible MAO-A inhibitor used as an anti-depressant in the clinic [70].

Like other MPACs such as PBT2 and clioquinol, M30 had moderate affinity for metal (Fe, Cu, and Zn) ions, but exhibited highly potent activity against iron-induced mitochondrial membrane lipid peroxidation, comparable to that of DFO (deferoxamine), a prototype iron chelator [71]. In pancreatic beta cell models, M30 markedly and dose-dependently inhibited H_2_O_2_-induced cytotoxicity, which is associated with decreased intracellular ROS formation and increased catalase activity [72].

The regulatory effects of M30 on APP and Aβ have been investigated both *in vitro* and *in vivo*. For example, *in vitro*, M30 significantly increased the release of soluble APPα into the conditioned medium and markedly lowered the secreted levels of Aβ in the conditioned medium [73]. *In vivo*, chronic treatment of aged mice with M30 (1 and 5 mg/kg; four times weekly for 6 months) showed significant positive impact on neuropsychiatry functions and cognitive age-related impairment [74]. In addition, M30 also significantly reduced cerebral Aβ level and iron accumulation in the treated mice [72]. In another study, chronic administration of M30 attenuated cerebral Aβ pathology and behavioral deficits in an APP/PS1 transgenic mice model of AD [75].

Neurogenesis activity of M30 has been examined in various cell culture and animal models. Several key *in vitro* activities include: (1) Decrease in cell death induced by serum deprivation and 6-hydroxydopamine in rat pheochromocytoma (PC12) cells [68]; (2) Increase in the expression levels of the transcripts of BDNF and growth-associated protein-43 (GAP43) in primary cortical cells [76]; (3) Protection against hydrogen peroxide or 3-morpholinosydnonimine-induced neurotoxicity in mouse NSC-34 motor neuron cells [73]. *In vivo* studies demonstrated that M30 enhanced mRNA expression levels of BDNF in the cortex and striatum and glial cell-derived neurotrophic factor (GDNF) in the hippocampus and spinal cord of mouse brain [77].

Taken together, the available evidence has shown that M30 possesses unique multiple properties, which make it potential useful medicine for AD therapy.

## 6. M30D: Second Generation M30 Analog

Brain network dysfunction in AD also involves basal forebrain cholinergic dysfunction, which is a consistent feature of AD. This dysfunction is closely related to the cognitive deficits observed in AD patients. Accordingly, restoring the dysfunctional cholinergic system has been first proposed as a rational approach to the treatment of AD. Indeed, the first and also the primary drugs approved by FDA for the symptomatic treatment of AD are AChEIs, which partly restore the cholinergic dysfunction by inhibiting AChE to increase the acetylcholine levels. In the clinic, AChEIs have been proved safe and effective in improving cognition and global functions in AD patients. Moreover recent studies also suggest that some currently marketed AChEIs may positively alter the course of AD, and slow down the disease progression. The disease-modifying potential of these AChEIs may due to the other mechanistic effects of these drugs, not directly related to AChE inhibition. For example, FDA approved anti-AD drug donepezil (AChE inhibitor) can reduce AChE-induced Aβ aggregation and fibrillogenesis *in vitro* by binding to the peripheral anionic site (PAS) of AChE [78]. Donepezil also shows potent activity (an IC_50_ of 0.170 μM) against BACE-1 (β-secretase), a transmembrane aspartic protease that cleaves the β-amyloid precursor protein resulting in Aβ peptide [79]. In theory, drugs that block BACE-1 could prevent Aβ accumulation, which may slow or stop AD progression. In addition, donepezil also interacts with sigma 1 (σ1) receptor within the same dose range as its anti-amnestic effects [80]. Furthermore, donepezil exhibits neuroprotective effects both *in vitro* and *in vivo* against a variety of neurotoxic insults including oxygen-glucose deprivation and glutamate-induced toxicity in primary cultures of rat cortical neurons, Aβ-induced toxicity in primary cultures of rat septal cholinergic neurons, SH-SY5Y cells or mice [81,82,83].

**Figure 5:**
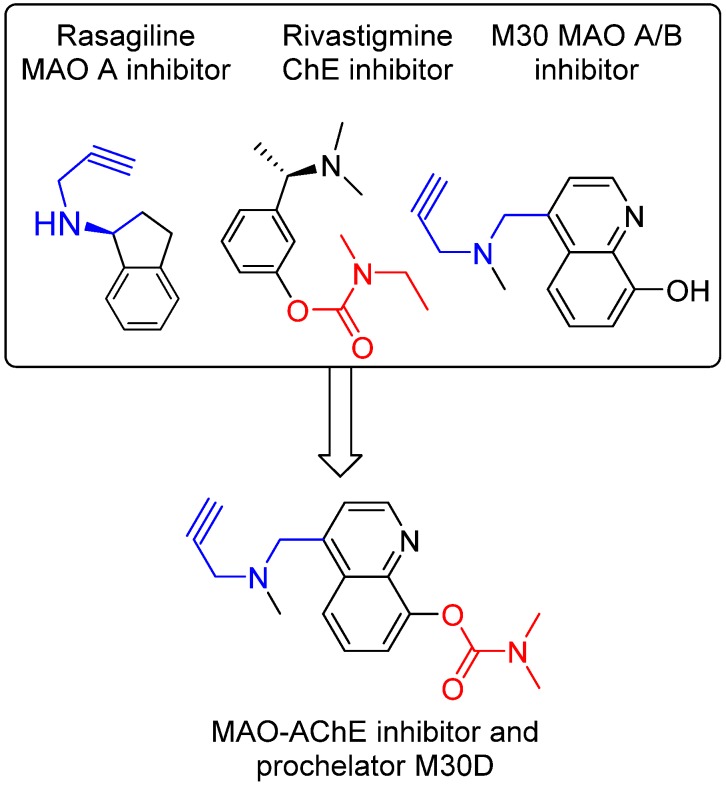
Design strategy resulting in multi-target ligand M30D, which contains propargylamine moiety (blue part) from anti-PD drug rasagiline and (dimethylcarbamoyl)oxy moiety (red part) from anti-AD drug rivastigmine.

The disease-modifying potential of AChEIs for AD therapy has encouraged the development of new AChE-based multitarget/network medicines, especially new medicines that can positively modulate the process of Aβ production and tau phosphorylation, but do not initiate AChE expression [84,85]. In accordance, we have developed a series of new AChE-based multi-target ligands derived from our lead M30 (Figure 5). A new lead M30D has been identified for further development. M30D contains the key pharmacophores of the FDA-approved anti-AD drug rivastigmine and acts as a prodrug of M30 (Figure 4). M30D was designed to target several key disease-related enzymes (proteins)/pathway of network dysfunction in AD, which include Aβ, Tau, AChE, MAO A/B, metal dyshomeostasis, oxidative stress, and inflammatory and neuroprotective pathways.

Preliminary studies have shown that M30D acted as a pseudo-irreversible AChE inhibitor such as rivastigmine to progressively inhibit AChE as the incubation time increased and reached maximum inhibition of AChE after 2 h pre incubation. However, unlike rivastigmine as a potent BuChE inhibitor, M30 D exhibited strong AChE inhibition with only minor activity against BuChE. In rat brain homogenates, M30D inhibited AChE and BuChE activity with the IC_50_ values of 0.5 ± 0.1 μM and 44.9 ± 6.1 μM, respectively, showing high selectivity (IC_50_ BuChE/IC_50_ AChE = 86.4) toward AChE inhibition. In addition, M30D was found to be highly potent MAO-A inhibitors with relatively weak activity against MAO-B, when measured in rat brain homogenates. The IC_50_ value for MAO A inhibition was 0.0077 ± 0.0007 μM, with activity about 3-fold more potent than M30. The IC_50_ value for MAO B inhibition was 7.9 ± 1.3 μM for M30D *vs*. 0.037 ± 0.02 μM for M30. Moreover, M30D was found to be a poor metal (Fe, Cu, and Zn) ion chelator *in vitro*, however, upon incubation with AChE, it exhibited high metal-binding affinity, similar to other 8-hydroxyquinol analogs, like VK28 [83]. Further studies suggested that *in vivo*, M30D acted as a pro-chelator, being metabolized to the active parental chelator M30 following pseudo-inhibition of AChE. In human SH-SY5Y neuroblastoma cells, M30D was found to have significant reduced toxicity compared to M30 [86]. Together, these preliminary data demonstrate the feasibility of our drug design strategy for rationally designing a new class of site-activated chelators with AChE and MAO-A and -B inhibitory activity.

## 7. *Ginkgo Biloba* (GB) Extract

*Ginkgo biloba* (GB) is a unique species of tree with various uses in traditional medicine. Both the nuts and the leaves of this tree have been used for centuries in Traditional Chinese Medicine for treating a number of diseases, including asthma, cough, and enuresis heart and lung dysfunctions and skin infections. Today, GB leaf extract is mainly used as a memory and concentration enhancer, and antivertigo agent. GB leaf extract is a really dirty drug, containing proanthocyanidins, terpenoids, flavonol glycosides, and other constituents [87]. It modulates a large number of drug targets and exhibits a variety of biological activities. For example, GB extract reversibly but nonselectively inhibits monoamine oxidase A/B and norepinephrine (NET), serotonin (SERT), dopamine (DAT) uptake transporters *in vitro* [88]. It inhibits platelet-activating factor and increase NO production in vessels, with subsequent effect on peripheral and cerebral blood flow [89,90]. In addition, GB leaf extract can act as a selective 5-HT1A receptor agonist *in vivo* [91]. It also displays antioxidant, neuroprotective and antiapoptotic properties, such as directly scavenging free radicals, indirectly blocking formation of free radicals, and inhibiting Aβ neurotoxicity [92,93,94].

EGb 761, a standardized extract formulation from GB leaf, has been extensively studied in terms of its effects on the cognition, especially as a possible treatment for AD. A comparison study in 2006 suggested that GB extract (160 mg daily dose) was as effective as a daily 5-mg dose of FDA anti-AD drug donepezil in the treatment of mild to moderate Alzheimer’s dementia [95]. However, a randomized controlled clinical trial with 176 participants in 2008 found that GB extract at a daily dose of 120 mg conferred no benefit in mild-moderate dementia over 6 months [96]. Similarly, a large, double-blind, randomized clinical trial found GB extract ineffective at preventing cognitive decline in older adults when given at a dose of 120 mg trice daily. This trial was conducted in six academic medical centers in the United States between 2000 and 2008, with 3,068 participants and a median follow-up of 6.1 years [97]. But another study in 2010 found that GB extract was significantly superior to placebo in the treatment of patients with dementia with neuropsychiatric symptoms. This study was conducted in multi-centre of 410 outpatients with mild to moderate dementia, with 240 mg of EGb 761 or placebo once daily for 24 weeks [98].

Recently a systematic review and meta-analysis were conducted based on three randomized controlled trials in patients with schizophrenia and eight randomised controlled trials in patients with dementia. The resulting data support the use of GB extract in patients with dementia and as an adjunctive therapy in schizophrenic patients [99]. In summary, although the positive results in AD animal models and in some clinical trials, the efficacy of GB extract in AD treatment still remains controversial. More studies are needed in order to shed more light on the effectiveness of GB extract for AD therapy.

## 8. Conclusions

Various processes and pathways are observed dysfunction in AD brain network, which involve Aβ accumulation and aggregation, tau hyperphosphorylation, cholinergic hypofunction, metal dyshomeostasis, glutamate excitotoxicity, cell cycle dysregulation and neuroinflammation, as well as disturbance of mitochondrial function and oxidative stress induced neurotoxicity. Moreover, these processes and pathways are found to overlap and influence one another in AD pathogenesis, which make understanding of cellular mechanisms of neurodegeneration a great challenge. This complex in AD pathogenesis has challenged the conventional and dominant paradigm in drug discovery, in which highly selective single-target drugs are screened for AD therapy. Network medicine, which employs holistic approaches to restore disease network dysfunction, may offer alternative hope for effectively fighting against AD. In fact, the fundamental principles from network medicine have been successfully applied to develop combination therapy or multi-target ligands for effective treatment of other complex diseases such as AIDS, cancer and depression. It is our hope that network medicine may also open up a new avenue for developing effective drugs for AD therapy.
